# Peptides derived from the dependence receptor ALK are proapoptotic for ALK-positive tumors

**DOI:** 10.1038/cddis.2015.102

**Published:** 2015-05-07

**Authors:** A Aubry, S Galiacy, L Ceccato, C Marchand, C Tricoire, F Lopez, R Bremner, C Racaud-Sultan, B Monsarrat, F Malecaze, M Allouche

**Affiliations:** 1 Université de Toulouse, UPS, EA4555, GR2DE, CPTP, Toulouse F-31300, France; 2 Lunenfeld Tanenbaum Research Institute, Mount Sinai Hospital, Toronto, M5G 1X5, Canada; 3 Department of Laboratory Medicine and Pathobiology, University of Toronto, Toronto, M5S 1A1, Canada; 4 CHU Purpan, Toulouse F-31300, France; 5 INSERM, UMR1037, CRCT, Toulouse F-31000, France; 6 INSERM, UMR 1043, CPTP, Toulouse F-31300, France; 7 CNRS, UMR 5282, CPTP, Toulouse F-31300, France; 8 CNRS, UMR 5089, IPBS, Toulouse F-31077, France

## Abstract

ALK is a receptor tyrosine kinase with an oncogenic role in various types of human malignancies. Despite constitutive activation of the kinase through gene alterations, such as chromosomal translocation, gene amplification or mutation, treatments with kinase inhibitors invariably lead to the development of resistance. Aiming to develop new tools for ALK targeting, we took advantage of our previous demonstration identifying ALK as a dependence receptor, implying that in the absence of ligand the kinase-inactive ALK triggers or enhances apoptosis. Here, we synthesized peptides mimicking the proapoptotic domain of ALK and investigated their biological effects on tumor cells. We found that an ALK-derived peptide of 36 amino acids (P36) was cytotoxic for ALK-positive anaplastic large-cell lymphoma and neuroblastoma cell lines. In contrast, ALK-negative tumor cells and normal peripheral blood mononuclear cells were insensitive to P36. The cytotoxic effect was due to caspase-dependent apoptosis and required *N*-myristoylation of the peptide. Two P36-derived shorter peptides as well as a cyclic peptide also induced apoptosis. Surface plasmon resonance and mass spectrometry analysis of P36-interacting proteins from two responsive cell lines, Cost lymphoma and SH-SY5Y neuroblastoma, uncovered partners that could involve p53-dependent signaling and pre-mRNA splicing. Furthermore, siRNA-mediated knockdown of p53 rescued these cells from P36-induced apoptosis. Finally, we observed that a treatment combining P36 with the ALK-specific inhibitor crizotinib resulted in additive cytotoxicity. Therefore, ALK-derived peptides could represent a novel targeted therapy for ALK-positive tumors.

Designing targeted therapy for cancer has been a major goal of the last decade. Oncogenic tyrosine kinases have raised early interest, because elucidation of their structure facilitated the development of small-molecule inhibitors with therapeutic efficiency.^1^ The pioneer BCR-ABL inhibitor molecule imatinib was approved for therapeutic use as early as 2001 to treat chronic myeloid leukemia and Ph1-positive acute lymphoblastic leukemia.^2^ Later on, inhibitors targeting receptors for epidermal growth factor or vascular endothelial growth factor were approved for treatment of solid tumors, such as lung and breast cancer. To date, many tyrosine kinase inhibitors (TKIs) are used in the clinic.^3^ However, cancers treated by TKIs invariably become resistant to therapy and relapse. Acquired resistance develops through various mechanisms including secondary mutations of the targeted oncogene or activation of alternative proliferative signaling pathways.^4^ It seems thus necessary to invent new strategies designed to attack the tumor on multiple fronts.

ALK (anaplastic lymphoma kinase) is an oncogenic receptor tyrosine kinase associated with many tumor types. *ALK* was first identified in 1994 as a rearranged gene fusion (*NPM–ALK*) resulting from the t(2;5)(p23;q35) translocation occurring in 75% human anaplastic large-cell lymphomas (ALCLs).^5, 6^ Other translocations or gene inversions involving *ALK* were later described in solid tumors including 50–60% inflammatory myofibroblastic tumors, and a small proportion of diffuse large B-cell lymphomas, breast and renal carcinomas.^7, 8^ Recently, 4–8% non-small-cell lung cancer (NSCLC) were found to harbor an echinoderm microtubule-associated protein-like 4 *(EML4)–ALK* fusion.^7, 9^ Resulting fusion proteins associate the N-terminal portion of a protein partner (containing in most cases a dimerization domain) to the entire intracellular portion of ALK, including its tyrosine kinase domain. Subsequent dimerization of this fusion protein leads to constitutive activation of ALK kinase, resulting in enhanced signaling for cell proliferation, survival and oncogenicity.^10^

The full-length *ALK* receptor cDNA codes for a transmembrane receptor tyrosine kinase of the insulin receptor superfamily, which is essentially expressed in the developing nervous system.^11, 12^ Some authors proposed the two heparin-binding factors pleiotrophin (PTN) and midkine as ligands for ALK.^10^ However, their binding to ALK is controversed and might be indirectly mediated by heparin.^13^ ALK kinase signaling most likely involves co-receptors and/or co-signaling molecules such as the transmembrane receptor tyrosine phosphatase beta/zeta (RPTPb/z), a receptor for PTN and midkine. In the absence of ligand, RPTPb/z dephosphorylates ALK, whereas PTN and midkine direct binding to RPTPb/z inactivates its phosphatase activity.^14^ Expression of the full-length ALK receptor was also observed in neuroblastoma, a pediatric tumor derived from the neural crest affecting the peripheral nervous system. The ALK kinase in neuroblastoma is most often constitutively active as a result of gain-of-function mutations or protein overexpression, due to *ALK* gene amplification or copy number increase.^10, 15^

ALK appears therefore as an interesting therapeutic target to treat ALK-positive tumors. Indeed, since the identification of NPM–ALK and other ALK fusions as oncogenes for ALCL and inflammatory myofibroblastic tumors,^6, 16, 17^ several pharmaceutical companies developed ALK-specific TKIs. In 2010, a TKI targeting ALK and c-MET, crizotinib^18^ (also called PF-02341066), was authorized in clinical trials as a second-line therapy for advanced stage NSCLC harboring EML4–ALK. The initial clinical responses were so encouraging that crizotinib is currently tested in a growing number of advanced ALK-positive tumors (clinicaltrials.gov). Nevertheless, the tumors invariably develop resistance to the inhibitor, mostly through mutations of the kinase active site.^19, 20^ Therefore, it appears necessary to design alternate treatments or to associate TKIs with other molecules. One promising strategy would be to impair distinct functions of the oncogenic tyrosine kinase through targeting different sites of the ALK protein.

We recently demonstrated that the ALK receptor tyrosine kinase belongs to the functional family of so-called ‘dependence receptors'.^21, 22^ Such dependence receptors function with a dual signaling: in the presence of ligand (or a situation mimicking a ligand, e.g., inducing receptor dimerization and activation), the receptor exerts a prosurvival/antiapoptotic effect on the cell; in contrast, in absence of ligand and when the cell is submitted to environmental or genotoxic stress, a dependence receptor becomes proapoptotic. The proapoptotic effect is mediated by caspase-dependent cleavage of the receptor, either releasing or exposing a proapoptotic domain/sequence (called ‘addiction/dependence domain' or ADD), thus amplifying the apoptotic process.^23^ Molecular analysis of ALK deletion mutants allowed us to map the ADD domain of ALK to a 36-amino-acid (aa) stretch located within the juxtamembrane intracytoplasmic region of ALK. The ADD of ALK lacks homology with any known protein motif implicated in apoptotic processes and is necessary for ALK proapoptotic function.^22^ The purpose of the present study was to design a novel targeted therapy, taking advantage of the proapoptotic function of ALK.

Our hypothesis was that a synthetic peptide could mimic the proapoptotic function of ALK. Therefore, we synthesized several peptides whose sequence reproduced the entire ADD domain (36 aa) of ALK or part of it (12 aa) to assay their effects on various tumor cell lines. We show that several of these ALK-derived peptides are proapoptotic for ALK-expressing, but not ALK-negative, tumor cells. In addition, the ALK-derived 36-aa peptide (P36) enhanced the cytotoxic effect of the ALK kinase inhibitor crizotinib in ALK-positive ALCL and neuroblastoma cell lines. Thus our results uncover a new strategy for targeting ALK-expressing tumors.

## Results

### Design of peptides derived from the ADD of ALK

**Figure 1:**
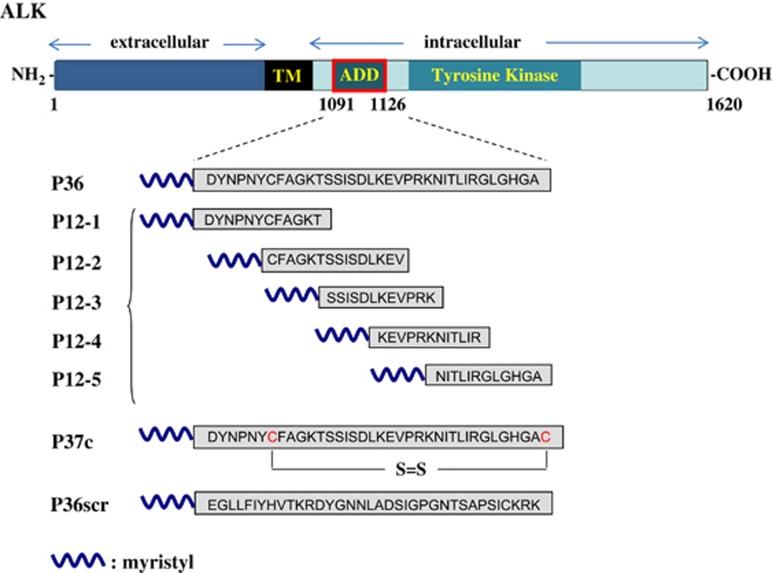
Design of peptides derived from the ADD (addiction/dependence domain) domain of ALK. The ALK receptor is composed of an extracellular, a transmembrane (TM) and an intracellular region with a tyrosine kinase domain. ALK has been identified as a dependence receptor, with both antiapoptotic and proapoptotic functions. An ADD has been localized in the juxtamembrane portion of the intracellular region (aa 1091–1126). Peptides of 36, 12 or 37 aa (circularized through an S–S bond) that mimicked the whole or partial sequence of the ADD domain were synthesized with a myristyl at their N terminus

All the peptides are schematically represented in Figure 1. We first designed a 36-aa peptide (P36), mimicking the whole ADD domain of ALK,^22^ to assay its potential to kill tumor cells. P36 was identical to the original ALK sequence 1091–1126 (accession: NP_004295.2), whereas P36scr (for ‘scrambled') served as a control. To stabilize P36 structure, a cyclic peptide (P37c) was also designed. It comprised P36 sequence with an additional C-terminal cysteine creating a disulfide bridge with cysteine 1097 within the sequence. Finally, to try and identify smaller active molecules, five overlapping peptides of 12 aa spanning the P36 sequence (P12-1 to P12-5) were synthesized. All the peptides were myristylated at the N terminus to facilitate cell penetration.^24^

### The ALK-derived peptide P36 is proapoptotic in ALK-positive ALCL and neuroblastoma cell lines

**Figure 2:**
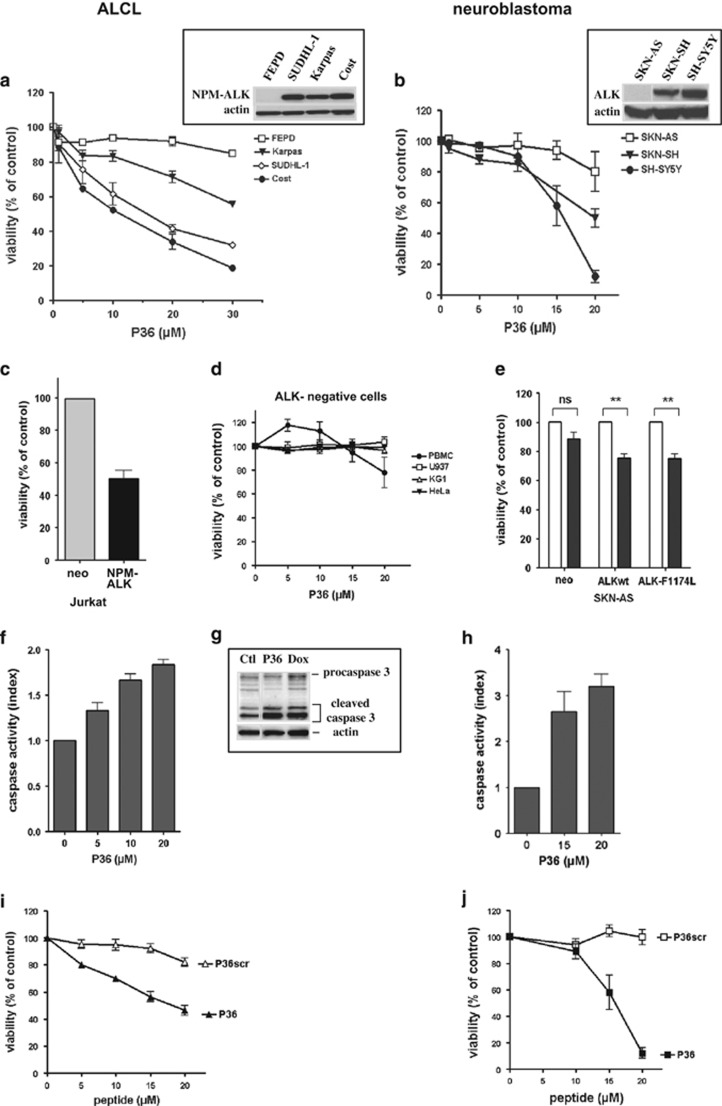
A peptide (P36) derived from the ADD domain of ALK specifically induces apoptosis in ALK-positive tumor cells. Cells were incubated with various concentrations of P36 for 48 h, or with culture medium without peptide for the control. Top panels: effect of P36 on the viability of ALK-positive and -negative ALCL (**a**) and neuroblastoma (**b**) cell lines. Insets: western blot analysis of ALK expression in the cell lines. Actin detection was used as a loading control. (**c**–**e**) Specificity of P36-induced apoptosis for ALK-expressing cells. ALK-negative normal (PBMC) or tumor (U937, KG1, HeLa) cells are insensitive to P36 (**d**). ALK-negative cells stably transfected with ALK variants (NPM–ALK in Jurkat (**c**), ALKwt or mutant ALK-F1174L in SKN-AS (**e**)) or an empty vector (neo) control were incubated with 5 *μ*M (Jurkat) or 15 *μ*M (SKN-AS) P36 and their viability assessed at 48 h. (**f**–**h**) P36 induces caspase-dependent apoptosis in ALK-positive cells. Cost ALCL (**f**) or SH-SY5Y neuroblastoma (**h**) cells were incubated with various doses of P36 for 48 h. Caspase 3/7 activity was measured and expressed as an index relative to control untreated cells (**f** and **h**). An increased caspase 3 cleavage was detected by western blot in Cost cells (**g**) treated for 6 h with P36 (10 *μ*M) or doxorubicin (Dox, 2 *μ*M) compared with culture medium (ctl). (**i** and **j**) Specificity of P36 cytotoxic effect on Cost ALCL (**i**) and SH-SY5Y neuroblastoma (**j**) compared with a scrambled version (P36scr) of P36. Values in all graphs represent the mean **±**S.E.M. from at least three independent experiments. NS, not significant, **P<0.01, statistical test: two-way analysis of variance (using Graphpad Prism 4 software)

The effect of P36 was first assayed on ALCL and neuroblastoma, which harbor cases with and without oncogenic ALK. Three ALCL lines carrying the NPM–ALK fusion (Karpas 299, SUDHL-1 and Cost) and one ALK-negative ALCL (FEPD) were incubated with increasing doses of P36 for 48 h. Similarly, two neuroblastoma lines carrying the ALK-F1174L mutation (SKN-SH, SH-SY5Y) and one ALK-negative neuroblastoma (SKN-AS) were treated with P36. In contrast to ALK-negative cells, ALK-positive ALCL and neuroblastoma cells responded to P36 in a dose-dependent manner, showing a decrease of the number of viable cells compared with their untreated control counterpart (Figures 2a and b). This ALK dependence of P36-induced cytotoxicity was further demonstrated in oncogenic ALK-transfected Jurkat^25^ and SKN-AS cells (Supplementary Figure 1), and other ALK-negative normal or tumor cells (Figures 2c–e).

Next, we sought to determine whether the decrease of viable cells induced by P36 was due to apoptosis. Incubation of Cost ALCL and SH-SY5Y neuroblastoma cells with P36 resulted in increased caspase 3/7 activity and cleaved forms of caspase 3, indicating that P36 induced caspase-dependent apoptosis in these cells (Figures 2f–h). P36 proapoptotic effect was sequence specific, as P36scr failed to affect P36-responsive cells (Figures 2I and j).

Taken together, our results indicate that the ALK-derived P36 peptide specifically induces apoptosis in ALK-expressing tumor cells.

### Effect of different ALK-derived peptides on cell viability

**Figure 3:**
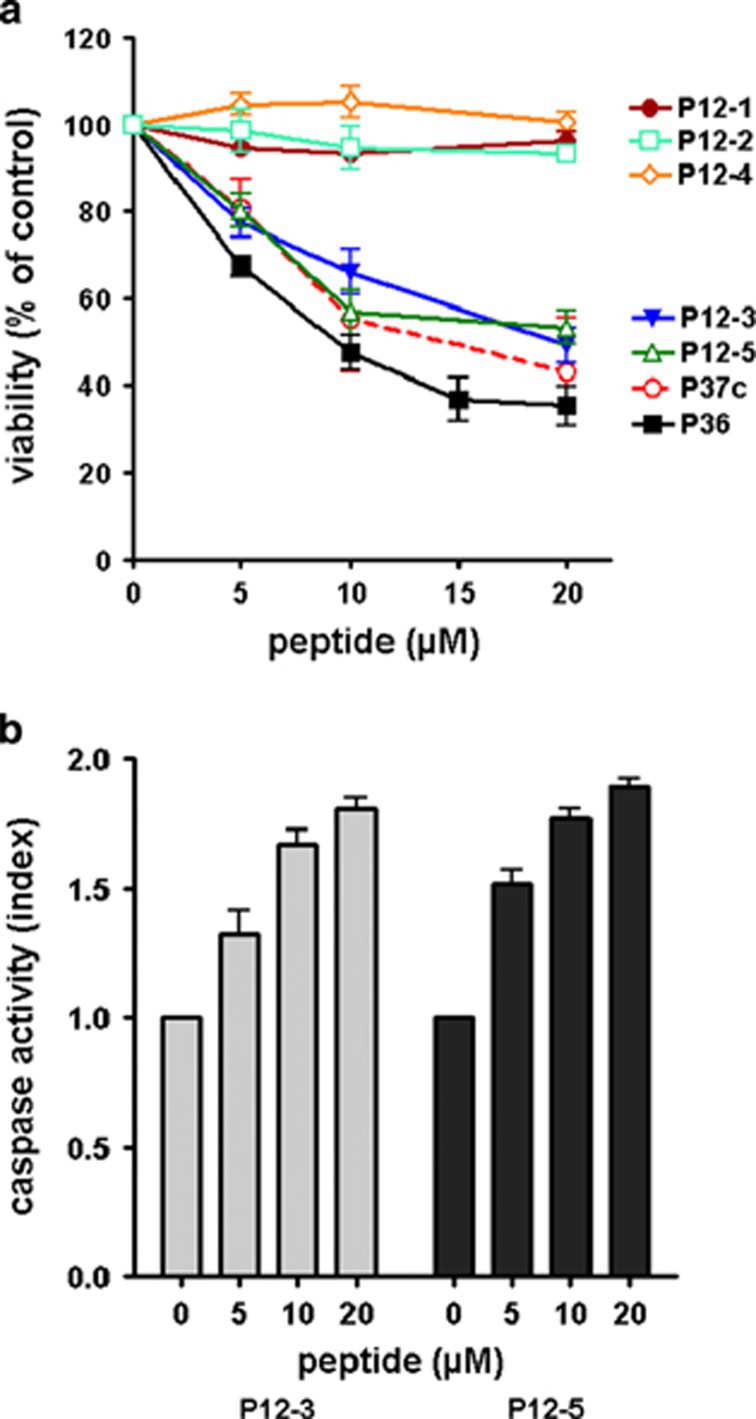
Cytotoxicity due to caspase-dependent apoptosis is induced by different peptides derived from the ADD domain of ALK. (**a**) Dose–response curves of different peptides on Cost cell viability. (**b**) Caspase 3/7 activity of P12-3 or P12-5-treated Cost cells. Results are the mean **±**S.E.M. from at least three independent experiments

To determine whether a smaller motif within the ADD of ALK could be proapoptotic, we tested the effect of five overlapping peptides of 12 aa (P12-1 to P12-5, Figure 1) on Cost cells. P12-3 and P12-5 displayed a dose-dependent cytotoxicity, whereas P12-1, P12-2 and P12-4 had no effect. The cyclic peptide, P37c, was also cytotoxic for Cost cells (Figure 3a). Similarly to P36, P12-3 and P12-5 increased caspase-dependent apoptosis (Figure 3b). However, we found P36 was slightly but significantly more effective than P12-3, P12-5 or P37c, respectively (*P*<0.05, Student's *t*-test, Figure 3a). This result led us to preferentially use P36 in further work.

### P36 peptide proapoptotic effect depends on myristoylation

**Figure 4:**
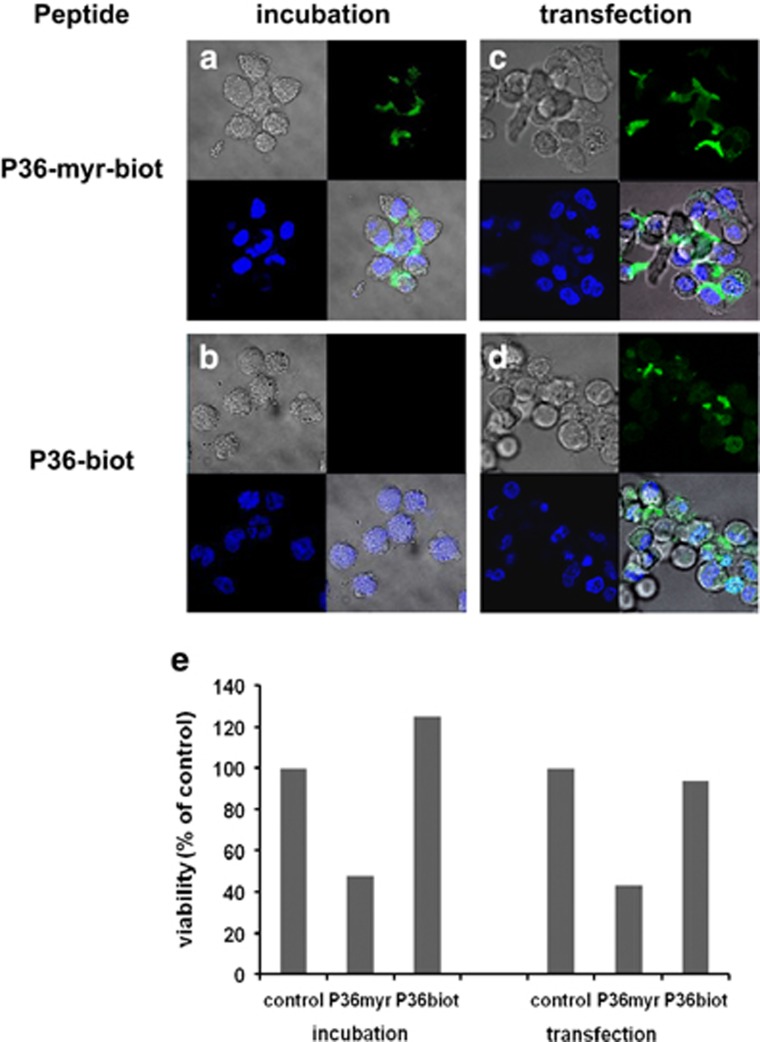
Myristoylation is required for P36 proapoptotic effect in ALK-positive tumor cells. Cost ALCL cells were either incubated 1 h at 37 °C (**a** and **b**) or transfected using an Amaxa Nucleofector device (**c** and **d**) with biotin-coupled P36, either *N*-myristylated (P36-myr-biot) or unmyristylated (P36-biot). The peptides were visualized with streptavidin-FITC after cell fixation and permeabilization. Four-panel composites show Nomarski interference contrast (gray), peptide (green), cell nuclei (DAPI, blue) or a merge of all three (bottom right panels). (**e**) Cell viability following incubation or transfection with peptides was assayed after 48 h of treatment. The graph represents one of two independent experiments

To determine the mechanisms implicated in P36-induced apoptosis, we first investigated whether P36 was internalized. Living cells were incubated for 1 h at 37 °C with a biotin-labeled *N*-myristylated P36 (P36-myr-biot), then fixed, permeabilized and stained with fluorescent streptavidin. A non-biotinylated peptide was used as negative control. P36-myr-biot was detected in the cytoplasm of both Cost ALCL (Figure 4a) and SH-SY5Y neuroblastoma cells (Supplementary Figure 2), but not in the nucleus. In contrast, cells incubated with a biotin-tagged non-myristylated P36 (P36-biot) did not display any fluorescence (Figure 4b), suggesting the myristyl was necessary for cell membrane insertion and penetration. To address this hypothesis, Cost cells were transfected with the biotin-coupled peptides using an Amaxa Nucleofector device and examined after 15 min incubation. With this alternate method, the non-myristylated P36-biot was delivered intracellularly as efficiently as P36-myr-biot (Figures 4c and d). To elucidate the respective roles of cell internalization and peptide myristoylation in the proapoptotic effect of P36, we compared the viability of cells treated with different peptides for 48 h following incubation or transfection. Results show that solely the myristylated P36 peptide was proapoptotic in both incubation and transfection conditions (Figure 4e). Of note, smaller myristylated ALK-derived peptides P12-3 (active) and P12-1 (inactive) were also internalized by cells (Supplementary Figure 2 and data not shown). These data demonstrate that the proapoptotic effect of active ALK-derived peptides depends on their myristoylation.

### P36 peptide proapoptotic effect is p53 dependent

To further explore the mechanism of P36-induced apoptosis, we conducted a search for P36 interaction partner proteins using surface plasmon resonance followed by mass spectrometry analysis. Biotinylated P36 was fixed onto a Biacore streptavidin-coated gold chip and cell protein extracts from Cost or SH-SY5Y peptide responder cells were allowed to bind onto the chip. Eluted bound proteins were analyzed by mass spectrometry. We identified a total of 13 proteins from Cost ALCL (Supplementary Table S1) and 179 proteins from SH-SY5Y neuroblastoma (Supplementary Table S2) that bound to P36, respectively. Assuming peptide cytotoxicity in ALCL and neuroblastoma cells might share a common mechanism, it seemed relevant to explore which P36-bound proteins were common to Cost and SH-SY5Y cells (Table 1). Six proteins were identical in both cell lines (i.e., SUB1, SSBP1, HRNR, XRCC5, EEF1A1 and LUC7L2). Interestingly, five of these structurally unrelated proteins have been reported to interact with the *p53* tumor suppressor gene or protein.

**Figure 5:**
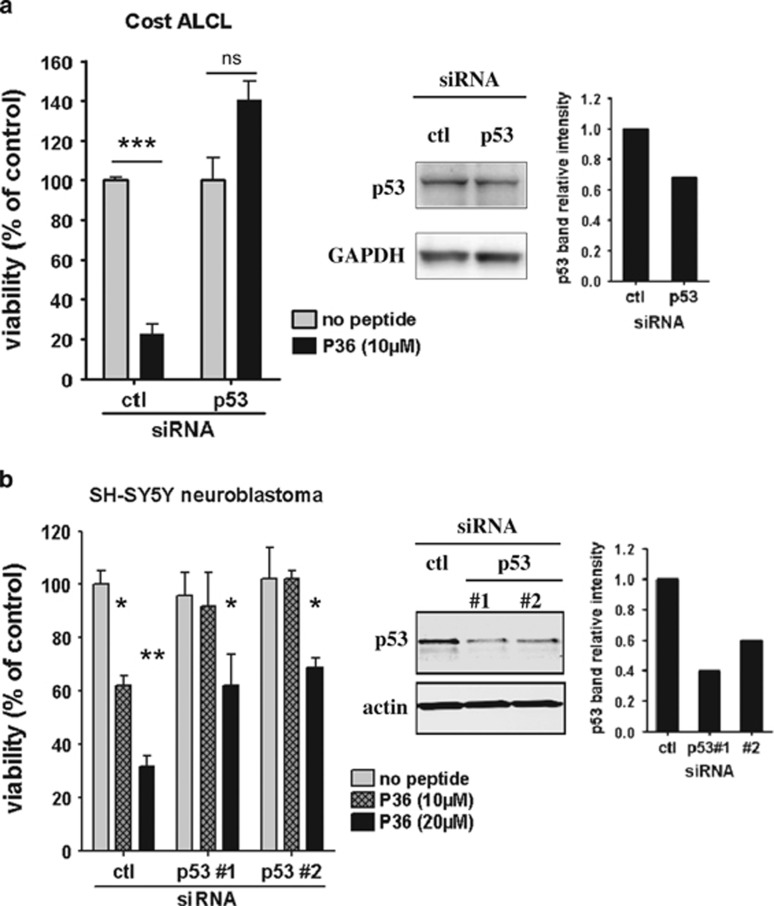
P36-induced apoptosis in ALK-positive tumor cells is p53 dependent. Cost ALCL (**a**) or SH-SY5Y neuroblastoma (**b**) cells were incubated with p53-specific (a pool of four siRNAs for Cost or two distinct siRNAs for SH-SY5Y) or non-targeted control pool (ctl) siRNAs for 48 h before addition of P36 or culture medium (no peptide) for another 48 h. Cell viability (left) and western blot analysis of p53 expression (middle) were assayed at that time. The intensity of p53 bands relative to loading controls (GAPDH, actin) was quantified by image analysis (graphs on the right). Results display one of two representative experiments with triplicate samples. Statistical test: Student's *t*-test. NS, non significant; *P<0.05; **P<0.01; ***P<0.001

We next asked whether p53 knockdown would affect P36-induced apoptosis in ALK-positive cells. Cost and SH-SY5Y were preincubated with p53-specific or control siRNAs for 48 h and then treated with P36 for a further 48 h. Preincubation of Cost ALCL with a pool of four p53-specific siRNAs only achieved a 30% reduction of p53 protein expression (Figure 5a, right panel), whereas in SH-SY5Y cells, two distinct siRNAs reduced the p53 expression level to 40% and 60% of control, respectively (Figure 5b, right panel). Although partial, p53 downregulation clearly rescued both Cost ALCL and SH-SY5Y neuroblastoma cells from P36-mediated cytotoxicity (Figures 5a and b). These results demonstrate that the proapoptotic activity of P36 is p53 dependent.

### Combined effects of P36 and crizotinib in ALK-positive tumor cells

**Figure 6:**
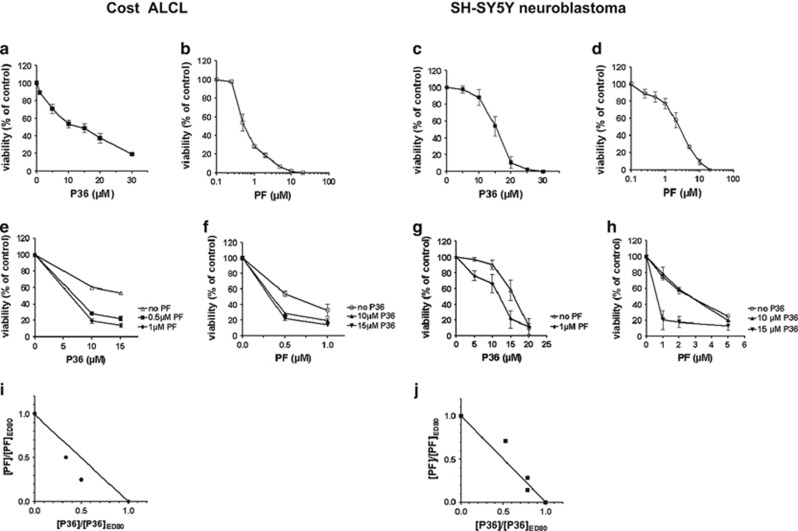
Combined cytotoxic effect of P36 and PF on ALK-positive tumor cells. Cost ALCL (left) or SH-SY5Y neuroblastoma (right) cells were incubated for 48 h with increasing doses of P36 (0–30 *μ*M) or PF (0–20 *μ*M) or a combination of both drugs as indicated. (**a**–**d**) Dose–response curves of P36 and PF on Cost (**a** and **b**) and SH-SY5Y (**c** and **d**) cell viability, respectively. (**e**–**h**) Viability curves for Cost (**e** and **f**) and SH-SY5Y (**g** and **h**) treated with a constant dose of either P36 or PF close to the IC_50_ while varying the dose of the other drug. (**i** and **j**) Normalized isobolograms drawn for ED_80_ indicate a synergistic trend (**i**, Cost) or an additive effect (**j**, SH-SY5Y) of P36 and PF combination

A promising therapeutic approach for ALK-expressing tumors is a targeted therapy using ALK kinase inhibitors. Crizotinib (PF-2341066, abbreviated ‘PF' in the text) is a dual kinase inhibitor for ALK and c-MET that is highly cytotoxic for ALK-positive ALCL cells *in vitro* and *in vivo.*^18^ To determine whether crizotinib (PF) and P36 could synergize in ALK-positive tumor cells, we examined the effect of single drug and combination treatments on the viability of Cost ALCL and SH-SY5Y neuroblastoma cells after 48 h exposure. To ensure an equivalent contributing effect from each drug, a dose–response was established for P36 and PF separately and the IC_50_ for each drug was determined (Cost cells: 10 *μ*M for P36 and 0.5 *μ*M for PF, respectively; SH-SY5Y cells: 16 *μ*M for P36 and 2.5 *μ*M for PF, respectively; Figures 6a–d). We then used a constant dose of either P36 or PF close to the IC_50_ while varying the dose of the other drug. Figures 6e–h show the combination of PF and P36 was always more efficient than each drug alone within the dose range assayed. To determine whether the effect of combined P36 and PF was synergistic or additive, normalized isobolograms were drawn (Figures 6i and j). In this type of analysis, points located on the straight line represent an additive effect for a given effective dose (ED), thus experimental data points that are above or under the straight line indicate an antagonist or synergistic effect, respectively.^26^ Figures 6i and j show at combined doses eradicating 80% of the viable cells (ED_80_), the killing effect of P36 and PF was synergistic in Cost ALCL and additive in SH-SY5Y neuroblastoma cells, respectively.

### P36 does not affect ALK phosphorylation

**Figure 7:**
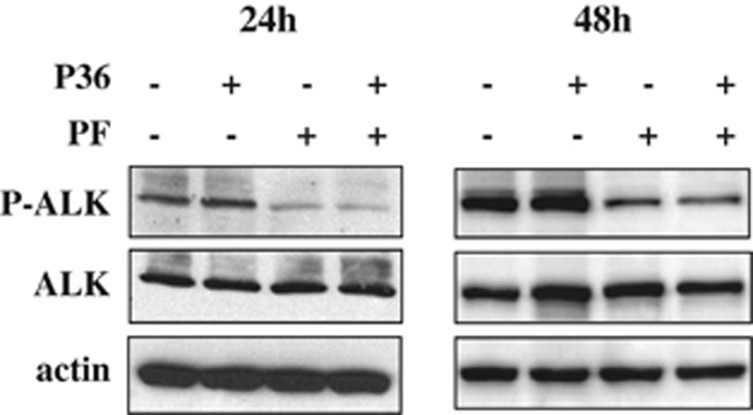
Different effects of P36 and PF on ALK phosphorylation. Western blot analysis of ALK phosphorylation in Cost cells treated for 24 or 48 h with P36 (10 *μ*M), PF (0.5 *μ*M) or both

The finding that P36/PF combination induced at least additive cytotoxicity in both ALCL and neuroblastoma cells suggested the two agents could act on different pathways. We thus examined the level of ALK tyrosine phosphorylation of Cost cells incubated for 24 or 48 h with P36, PF or both drugs (Figure 7). As expected, the kinase inhibitor PF decreased ALK phosphorylation. In contrast, cell treatment with P36 did not affect the level of ALK phosphorylation. Moreover, combining PF and P36 did not enhance PF-induced dephosphorylation. Note that the expression level of total ALK protein during P36 treatment did not vary, indicating the protein was not degraded. Taken together, our results indicate the mechanism of P36 cytotoxicity is not mediated by kinase inhibition.

## Discussion

ALK drives tumorigenesis in several types of cancers, including ALCL and neuroblastoma. ALK-targeted therapy using specific inhibitors such as crizotinib has shown promising results, but inevitably associates with resistance. To overcome this issue, we devised a strategy based on the dependence receptor properties of ALK, implying that in the absence of ligand or kinase activation, ALK is proapoptotic.^21, 22^ Using synthetic peptides to mimic the ALK proapoptotic domain (ADD),^22^ we showed human ALCL and neuroblastoma tumor cells were killed in a dose-dependent manner following incubation with an ADD-like peptide (P36). Cell death was due to caspase-dependent apoptosis. Cell sensitivity to the peptide was dependent on ALK expression by tumor cells as: (i) ALK-negative tumor cells failed to respond and (ii) expressing either NPM–ALK or full-length ALK restored P36-responsiveness of lymphoma or neuroblastoma cells, respectively. Unfortunately a loss-of-function approach, that is, silencing ALK by siRNA to confirm the requirement of ALK expression for P36-mediated apoptosis was not applicable. Indeed, ALK-expressing tumors present an oncogene addiction and die by apoptosis following ALK downregulation.^27, 28^ Moreover, despite different subcellular localization of oncogenic ALK forms (nuclear and cytoplasmic for NPM–ALK,^25^ cytoplasmic and at the plasma membrane for ALK,^29^ respectively) (Supplementary Figure 1), P36 killed both ALCL and neuroblastoma cells. Worthy of note, P36 was not toxic for normal peripheral blood mononuclear cells (PBMCs), which do not express ALK, making P36 a potential therapeutic agent.

Peptides are an attractive therapy due to relatively easy synthesis. To minimize protease degradation, cyclization can be used to strengthen peptide structure. Here, we showed P37c, like P36, was proapoptotic. Another challenge resides in delivering peptides at the right site, that is, tumor cells. We chose to add an *N*-myristyl tag because it reportedly facilitates insertion in the plasma membrane and subsequent peptide translocation into cells.^24, 30^ Surprisingly, the *N*-myristyl was not only necessary for cellular uptake but also for the cytotoxic activity of ALK-derived peptides. Note that many cell signaling molecules are *N*-myristylated. Myristoylation directs these proteins to specific cell compartments (e.g., the Golgi apparatus, endoplasmic reticulum or mitochondria), impacting their function.^31^ Moreover, the proapoptotic domain of ALK receptor is located close to the plasma membrane. Thus, *N*-myristoylation likely has a role in peptide subcellular localization and proapoptotic signaling. However, *in vivo* peptide delivery may require additional coupling, for example, to nanoparticles,^32^ and we may consider this approach in further work.

To our knowledge, this is the first time that synthetic peptides derived from the ADD domain of a dependence receptor are used to target and kill tumor cells. Alternative approaches using the proapoptotic potency of dependence receptors are also currently developed. In aggressive tumors coexpressing netrin-1 and its receptors DCC and UNC5H, a double therapeutic approach was designed: (i) overexpression of the intracellular domain (including the ADD) of the receptors, and/or (ii) synthesis of soluble decoy receptors used as ligand traps for autocrinally secreted netrin.^33, 34, 35^ Both strategies interfere with netrin-1 ability to inhibit DCC- or UNC5H-induced cell death. These treatments killed netrin-1-expressing tumor cells both *in vitro* and *in vivo*. However, the latter approach is only feasible for a receptor expressed on the cell surface, which is not the case for NPM–ALK or other ALK-associated fusions, therefore justifying our approach to use peptides designed to enter tumor cells.

Some authors have designed hybrid molecules coupling penetrating peptides derived from *tat* or *antennapedia* to proapoptotic nonspecific peptides such as BCL-2 family domain 3-mimetics.^36^ The originality of ALK-derived peptides described in this report is their specificity for ALK-expressing tumors. It would certainly be interesting to develop ALK-ADD peptide-mimetic small molecules in the future.

The fact that P36 only killed ALK-expressing cells suggested the peptide would likely interfere with ALK signaling. P36 may either antagonize ALK dependence receptor antiapoptotic or enhance proapoptotic signaling. Perhaps P36-mediated cytotoxicity requires binding of endogenous ALK. The chaperone Hsp90 was reported to protect ALK from degradation by the proteasome.^37, 38^ On the basis of the detection of Hsp90 in the proteomic analysis (Supplementary Table S2), P36 might sequester Hsp90 thus favoring ALK degradation and apoptosis. However, the lack of evidence for ALK degradation upon P36 cell treatment (Figure 7) did not support this hypothesis. A more likely mechanism would imply the titration of regulatory adaptors bound to the proapoptotic ALK domain. These propositions need to be further elucidated.

Cross-analysis of the P36 interactome of ALCL and neuroblastoma cells yielded six proteins susceptible to interact with the tumor suppressor p53, whereas the remaining proteins were involved in pre-mRNA splicing.

The presence of a significant number of pre-mRNA splicing factors among P36-interacting proteins was striking. Alternative splicing is an important regulator of apoptosis, as it determines the balance of antiapoptotic and proapoptotic isoforms of numerous gene products including caspases and members of the BCL-2 and p53 families.^39^ Therefore, sequestration of splicing factors through interaction with P36 could possibly impair the balance of antiapoptotic and proapoptotic signals.

Another feature was the ability of several P36-interacting proteins to interact with the tumor suppressor p53 or regulate its expression.^40, 41, 42, 43, 44, 45, 46, 47^ These interactions could modulate p53-dependent apoptosis or DNA damage response at the mitochondrial and/or nuclear level, respectively. Our finding that p53 downregulation (although partial) could rescue ALK-positive cells validated the role of p53 in P36-induced apoptosis. Moreover, P36 (in the absence of siRNA) did not increase p53 protein expression (data not shown), suggesting P36-induced apoptosis may involve a p53-dependent mitochondrial pathway.^48^

An interesting protein in our proteomic analysis was SUB1(PC4), a multifunctional nuclear protein having important roles in DNA transcription, replication and repair.^49^ PC4 interacts with p53 and is also regulated by p53. A recent study reported PC4 protein was upregulated in NSCLC carcinoma tissues. Moreover, PC4 knockdown induced growth arrest and apoptosis of NSCLC cell lines.^50^ Thus PC4 could act as an antiapoptotic protein. In the present report, we speculate P36 could sequester PC4 in the cytoplasm thus preventing it to interact with its nuclear target p53.

The recent development of ALK-specific TKIs brought hope for chemotherapy-resistant or relapsing ALK-expressing tumors. Crizotinib treatment could extend disease-free survival for ALK-positive NSCLC, ALCL and neuroblastoma.^51, 52, 53^ However, TKI resistance developed mostly due to secondary mutations.^4, 54^ Interestingly, acquired mutations affected the same hotspots (e.g., F1174) as those described in primary neuroblastomas.^19, 20^ Herein, we showed the F1174L-expressing SH-SY5Y and transfected SKN-AS neuroblastoma cells were sensitive to P36. This is in contrast with the reported resistance of this mutant to crizotinib.^20, 55^ Along the same line, we observed a fivefold difference between the IC_50_ of PF in SH-SY5Y neuroblastoma *versus* Cost ALCL, whereas the IC_50_ of P36 was in the same range for both cell lines (Figure 6).

Taken together, these observations brought evidence to associate two ALK-specific targeted drugs, P36 and PF, to enhance cytotoxicity. Whereas TKI-mediated killing implied inhibition of ALK phosphorylation, P36 cytotoxic effect involved a different, although presently unknown, mechanism. Accordingly, we observed an additive effect of TKI/ P36 combination in both ALK-positive ALCL and neuroblastoma.

Collectively our findings are novel and should contribute to better understand the mechanism of P36 proapoptotic activity. The proteomic analysis uncovered a role for p53, and further studies are in progress to establish which proteins have a direct or indirect role in the proapoptotic effect of P36 and how they act.

In conclusion, peptides derived from the ADD domain of ALK may represent a new therapeutic approach in ALK-expressing tumors. These peptides could potentially be useful both in tumors expressing ALK fusion proteins and the full-length ALK receptor. The enhancement of P36-mediated cytotoxicity when in association with an ALK TKI could be especially valuable in targeting tumors bearing ALK rearrangements or activating mutations acquired following a first-line treatment.

## Materials and Methods

### Reagents

All chemicals were from Sigma (www.sigmaaldrich.com) and culture reagents from Gibco, (www.invitrogen.com).

### Peptides

Peptides of 36 (P36 and P36scr), 37 (P37c) and 12 (P12-1, P12-2, P12-3, P12-4, and P12-5) aa, respectively, were synthesized by 9-fluorenylmethyloxycarbonyl (Fmoc) solid-phase chemistry by Covalab (Villeurbanne, France) and purified by reverse-phase high-performance liquid chromatography (HPLC). Their purity (75–95%) was verified by HPLC and mass spectrometry. All the peptides (unless mentioned) were synthesized with a myristyl group at the N terminus and their C terminus was amidated. For visualization studies, peptides were coupled to biotin at the C terminus. Stock solutions of peptides were prepared in distilled water at 2.5 mM (P36, P36scr, P37c), 5 mM (P12-3, P12-4, P12-5) or in DMSO at 20 mM (P12-1, P12-2), sonicated, aliquoted and stored at −80 °C.

### ALK inhibitor

Racemic PF-2341066 [3-[1-(2,6-dichloro-3-fluoro-phenyl)-ethoxy]-5-(1-piperidin-4-yl-1*H*-pyrazol-4-yl)-pyridin-2-ylamine] (crizotinib) was synthesized according to the method described in the patent international application WO 2006/021881.

### Cell culture

Cells were cultured at 37 °C in a humidified atmosphere containing 5% CO_2_. SU-DHL1, Karpas 299, Cost and FEPD ALCL cell lines^56, 57, 58, 59^ were maintained in Iscove's modified Dulbecco's medium (IMDM) (Gibco, www.invitrogen.com), 15% fetal bovine serum (FBS), 100 U/ml penicillin, 100 μg/ml streptomycin, 2 mM glutamine, and 1 mM sodium pyruvate. The Jurkat (clone E6.1; TIB-152 from ATCC, Rockville, MD, USA) human T-lymphoblastic, KG1 and U937 myeloblastic leukemia cell lines were maintained in RPMI 1640 containing 10% FBS, 2 mM glutamine, 100 U/ml penicillin, 100 μg/ml streptomycin, and 1 mM sodium pyruvate. Transfected Jurkat cells, that is, Jurkat/neo and Jurkat/NPM–ALK cells^25^ were cultured in the continuous presence of 2 mg/ml G418. The HeLa uterine carcinoma cell line was maintained in Dulbecco's modified Eagle's medium (DMEM) (Gibco), 10% FBS, 100 U/ml penicillin, 100 μg/ml streptomycin, 2 mM glutamine, and 1 mM sodium pyruvate. Neuroblastoma cell lines SKN-SH (ATCC: HTB-11), SHSY-5Y (ATCC: CRL-2266) and SKN-AS (ATCC: CRL-2137) were cultured in RPMI 1640, 10% FBS, 100 U/ml penicillin, 100 μg/ml streptomycin, 2 mM glutamine, and 1 mM sodium pyruvate. PBMC from five healthy donors isolated using a Ficoll gradient were kindly provided by Dr Nabila Jabrane-Ferrat (CPTP, INSERM U1043, Toulouse, France) and used as normal lymphoid cell controls.

### Cell transfection

SKN-AS cells were stably transfected using Fugene HD (Promega, www.promega.com) with pcDNA3.1 (empty vector), pcDNA-ALKwt or pcDNA-ALK-F1174L,^15^ cloned by limiting dilution and cultured in the continuous presence of 0.5 mg/ml G418.

### Western blot

Cells were washed once with PBS, pelleted, and extracted in RIPA buffer (150 mM NaCl, 20 mM Tris-HCl, pH 7.7, 4 mM EDTA, 0.5% Triton X-100) containing 10 μg/ml leupeptin, 2 μg/ml aprotinin, 1 mM 4-2-aminoethyl-benzenesulfonyl fluoride, 1 mM sodium orthovanadate, and 4 mM sodium fluoride for 30 min on ice. Cell extracts were either centrifuged at 10 000 × *g* for 20 min at 4 °C and the supernatant recovered, or sonicated for better extraction of nuclear proteins. The protein content was quantified using the Bio-Rad (www.biorad.com) Bradford protein assay. For western blotting, total cell proteins were subjected to SDS-polyacrylamide gel electrophoresis (SDS-PAGE) in a 4–12% bis/tris acrylamide (Novex NuPAGE, Invitrogen) gradient gel using the Nupage system with Mops SDS buffer under reducing conditions and transferred for 3 h at 60 V onto a nitrocellulose membrane. The blots were hybridized with the following primary antibodies:

ZAL4 anti-human ALK rabbit monoclonal antibody (1/200, Invitrogen), phospho-ALK (Y1604) (1/1000, #3341, Cell Signaling Technology, www.cellsignal.com) rabbit antiserum, mouse monoclonal anti-caspase 3 (1/200, sc-7148, Santa Cruz Biotechnologies, www.scbt.com), rabbit polyclonal anti-p53 (1/500, #554167, BD Pharmingen, www.bdbiosciences.com) and rabbit polyclonal anti-GAPDH (1/2000, Millipore, www.millipore.com) antibodies. Secondary antibodies were horseradish peroxidase-coupled anti-rabbit (1/1000) or anti-mouse (1/1000) Ig antiserum from Cell Signaling. For beta-actin detection, a peroxidase-coupled mouse monoclonal antibody (1/7500, Sigma) was used. Signal detection was performed with an enhanced chemoluminescence kit (ECL, Amersham, www.gelifesciences.com).

### Cell viability assay

Exponentially growing cells were incubated in 96-well white microplates with a clear bottom at 10^5^ cells/ml with peptide, PF, both, or culture medium for control, for 48 h at 37 °C. For adherent cell lines, the microwells were pre-coated with 50 *μ*g/ml poly-l-lysine for better adhesion and cells were plated 24 h prior to treatment. At the end of incubation, cell viability was assessed by measuring the ATP content of wells using the luminescence-based CellTiterGlo assay (Promega) with a Berthold Mithras (http://www.berthold.com) plate reader. Each culture condition was assayed in triplicate.

### Apoptosis assay

Exponentially growing cells were incubated in 96-well microplates at 10^5^ cells/ml with peptide, PF or both, or culture medium for control, for 48 h at 37 °C. Apoptosis was assessed by measuring the relative caspase activity of treated *versus* control cells using the luminescence-based Caspase 3/7 Glo assay (Promega). Each culture condition was assayed in triplicate.

### Peptide penetration assay

To enable visualization, we used C-terminal biotin-tagged peptides. Exponentially growing Cost cells were incubated for 1 h at 37 °C with 10 *μ*M peptide. Alternatively, the peptides were electroporated into Cost cells with an Amaxa Nucleofector II device using program A30 and Nucleofector solution V (Lonza, www.lonza.com), according to a customized manufacturer protocol^60^ after which the cells were incubated in IMDM+1% FBS for 15 min at 37 °C. Then cells were centrifuged at 300 × *g* for 5 min, fixed, permeabilized and incubated with fluorescein isothiocyanate-coupled streptavidin (BD Pharmingen, www.bdbiosciences.com, 1/400) at room temperature using the Dako (www.dako.com) Intrastain kit. After washing and a second fixation in PBS-formaldehyde 1%, cells were mounted on slides and examined using an LSM 510 confocal microscope (Zeiss Axiovert 100, Carl Zeiss France, Marly le Roi, France).

### Detection of surface plasmonic resonance and mass spectrometry

Real-time binding experiments were performed with a BIAcore 3000 biosensor instrument (www.gelifesciences.com) and quantified in terms of resonance units (RU) (1000 RU=1ng of protein bound/mm^2^ of flow cell surface).^61^ Synthetic biotinylated peptides were immobilized onto streptavidin-coated carboxymethylated dextran Biacore Sensor chips (www.gelifesciences.com). Cell extracts (100 μg/ml) were injected in the running buffer (HEPES 10 mM, NaCl 150 mM, EDTA 3 mM, polysorbate 0.005%). For recovery experiments, an optimized protocol was applied.^62, 63^ Synthetic P36 biotinylated peptide was immobilized at a level of 2000 RU. Cell extracts from Cost or SH-SY5Y were injected at 500 μg/ml. Five recovery cycles were performed for each cell line to reach a total amount of 2000 RU of recovered proteins. Further analysis of the recovered proteins was performed by nano-LC–MS/MS using an Ultimate3000 system (Dionex, Amsterdam, The Netherlands) coupled to an LTQ Orbitrap XL mass spectrometer (Thermo Fisher Scientific, Bremen, Germany).^62, 63^

### RNAi transfection

RNAi transfection was optimized for Cost and SH-SY5Y cells therefore achieved by different methods and reagents. Cost cells were incubated for 48 h with 1 *μ*M Accell Human TP53 siRNA SMARTpool or control Accell Non-targeting Pool siRNAs (Dharmacon, http://dharmacon.gelifesciences.com) in a 1 : 1 vol/vol mix of Accell siRNA Delivery Media and IMDM containing 1% FBS, according to the manufacturer's protocol. SH-SY5Y cells were seeded in poly-l-lysine pre-coated six-well plates and preincubated overnight in RPMI containing 1% FBS in the absence of antibiotics. The next day, SH-SY5Y cells were transfected using Dharmafect-1 transfection reagent (Dharmacon) with one of two siRNAs targeting p53 (Dharmacon, siGenome si-p53 #1: D-003329-05, si-p53 #2: D-003329-07) at 50 nM or an siRNA control (Qiagen, www.qiagen.com: Allstar negative control, cat# SI03650318). For both Cost and SH-SY5Y, cell incubation with siRNAs lasted for 48 h to allow p53 gene downregulation. Then P36 was added at 10 or 20 *μ*M for the further 48 h. Cell viability and p53 expression (western blot) were assessed at that time.

### Statistical analysis

Statistical analysis was done using the Student's *t*-test and two-way analysis of variance using GraphPad Prism 4 software (www.graphpad.com/scientific-software/prism/).

## Figures and Tables

**Table 1:** List of P36-interacting proteins that are identical or similar in Cost ALCL and SH-SY5Y neuroblastoma cell lines

*Cell lines*	*Protein symbol*	*Cell process and function*
*Interaction with the p53 gene/protein or p53-dependent signaling pathways*		
Cost and SH-SY5Y	SUB1 (PC4)	Transcriptional coactivator for p53, neural gene silencing
Cost and SH-SY5Y	SSBP1	p53-dependent DNA damage
Cost and SH-SY5Y	HRNR	Skin protein, member of S100 family, elevated in various tumors
Cost and SH-SY5Y	XRCC5 (KU80)	RNA helicase, repair of DNA ds breaks by NHEJ and V(D)J recombination
Cost and SH-SY5Y	EEF1A1	Elongation factor, protein biosynthesis
		
*Regulation of pre-mRNA splicing*		
Cost and SH-SY5Y	LUC7L2	Pre-mRNA splicing, recognition of non-consensus splice donor sites
Cost	LUC7L3	Pre-mRNA splicing, formation of spliceosome
Cost	SFRS3	Pre-mRNA splicing, neural differentiation, proto-oncogene
Cost	SFRS11	Pre-mRNA splicing, neural differentiation
Cost	DDX46	RNA helicase, pre-mRNA splicing
SH-SY5Y	DDX5	RNA helicase, p53-dependent DNA damage and apoptosis, neural differentiation
SH-SY5Y	DDX39	RNA helicase, pre-mRNA splicing
SH-SY5Y	SFRS2	Pre-mRNA splicing, cisplatin-mediated apoptosis
SH-SY5Y	SFRS7	Pre-mRNA splicing, neural gene silencing
SH-SY5Y	U2AF1	Pre-mRNA splicing, spliceosome
SH-SY5Y	U2AF2	pre-mRNA splicing, spliceosome

P36-interacting proteins were classified in two functional families: proteins potentially interfering with p53 tumor suppressor, and mRNA splicing factors. Information on genes' function was retrieved from Genecards (www.genecards.org)

## Abbreviations

ALK: anaplastic lymphoma kinase
TKI: tyrosine kinase inhibitor
NPM: nucleophosmin
ALCL: anaplastic large-cell lymphoma
NSCLC: non-small-cell lung cancer
EML4: echinoderm microtubule-associated protein-like 4
PTN: pleiotrophin
RPTPb/z: receptor tyrosine phosphatase beta/zeta
ADD: addiction/dependence domain
aa: amino acid
PBMC: peripheral blood mononuclear cells
PF: PF-02341066 or crizotinib
ED: effective dose
HPLC: high-performance liquid chromatography
IMDM: Iscove's modified Dulbecco's medium
FBS: fetal bovine serum
DMEM: Dulbecco's modified Eagle's medium
SDS-PAGE: SDS-polyacrylamide gel electrophoresis
RU: resonance units
