# Supplementary material for: Peptides derived from the dependence receptor ALK are proapoptotic for ALK-positive tumors
Source: Cell Death Dis. 2015 May 7;6(5):e1736–. doi: 10.1038/cddis.2015.102 (PMC4669685; doi:10.1038/cddis.2015.102)
Supplement: Supplementary Information [file cddis2015102x1.pdf]

SUPPLEMENTARY INFORMATION

Legends

Supplementary Figure 1

Immunofluorescence detection of ALK in SKN-AS neuroblastoma cells transfected with control vector (neo), ALKwt or ALK-F1174L mutant cDNA using ZAL4 anti-ALK antibody followed by a FITC-coupled anti-rabbit Ig antibody. Note a cytoplasmic localization in both wt and mutant ALK-expressing cells, with a particular reinforcement at the membrane in ALKwt-transfected cells.

Supplementary Figure 2

Evidence of intracellular penetration of myristylated P36 and P12-3 peptides in SH-SY5Y neuroblastoma and Cost ALCL tumor cells. Cells were incubated for 1 h at 37°C with biotin-coupled, myristylated P36 (P36-myr-biot) or P12-3 (P12-3-myr-biot) peptides, then fixed, permeabilized and stained with streptavidin-FITC, as indicated. The peptides (in green) localize in the cytoplasm, especially close to the plasma membrane and in structures resembling the Golgi apparatus.

Supplementary Tables

Table S1: List of P36-interacting proteins from the Cost ALCL cell line

Table S2: List of P36-interacting proteins from the SH-SY5Y neuroblastoma cell line
