# Supplementary figures and images for: Peptides derived from the dependence receptor ALK are proapoptotic for ALK-positive tumors
Source: Cell Death Dis. 2015 May 7;6(5):e1736–. doi: 10.1038/cddis.2015.102 (PMC4669685; doi:10.1038/cddis.2015.102)

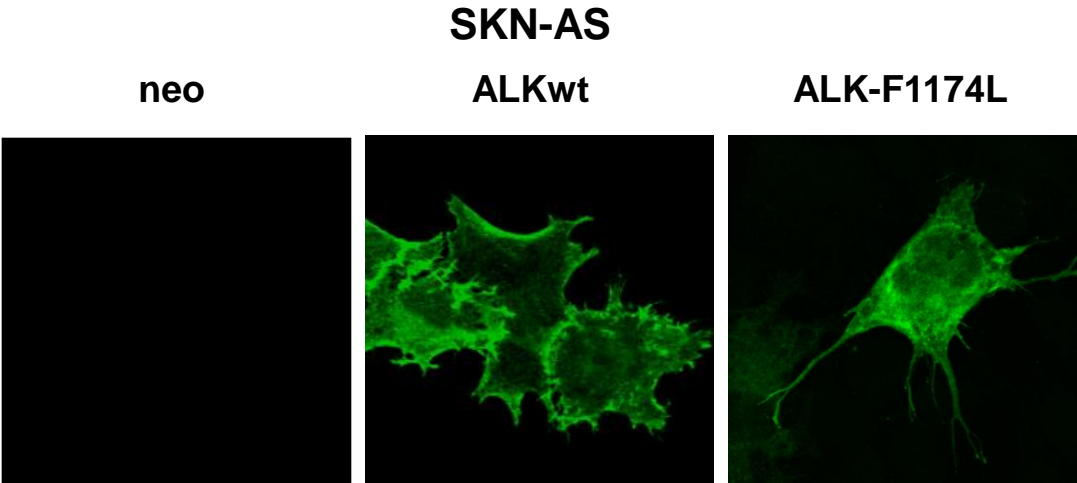

Supplement: Supplementary Figure 1 [file cddis2015102x2.pdf]

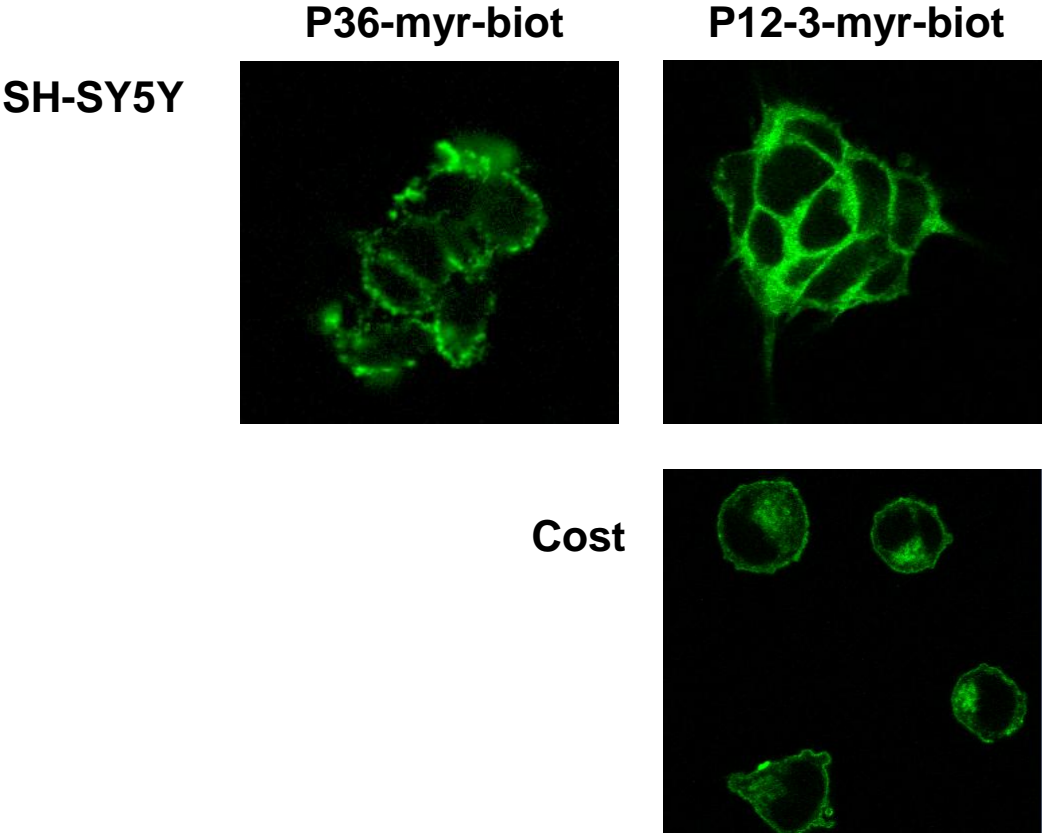

Supplement: Supplementary Figure 2 [file cddis2015102x3.pdf]
