# Supplementary material for: Peptides derived from the dependence receptor ALK are proapoptotic for ALK-positive tumors
Source: Cell Death Dis. 2015 May 7;6(5):e1736–. doi: 10.1038/cddis.2015.102 (PMC4669685; doi:10.1038/cddis.2015.102)
Supplement: Supplementary Table S2 [file cddis2015102x5.pdf]

Table S2 : List of P36-interacting proteins from the SH-SY5Y neuroblastoma cell line

Hit	ID	Description	Score	MW	Peptides
1	IPI00644712	XRCC6 X-ray repair cross-complementing protein 6 (Ku70)	683,26	69799	29
2	IPI00414676	HSP90AB1 Heat shock protein HSP 90-beta	604,36	83212	25
3	IPI00784154	HSPD1 60 kDa heat shock protein, mitochondrial	825,1	61016	25
4	IPI00021439	ACTB Actin, cytoplasmic 1	508,6	41710	25
5	IPI00418471	VIM Vimentin	461,23	53619	26
6	IPI00011107	IDH2 Isocitrate dehydrogenase [NADP], mitochondrial	421,91	50877	23
7	IPI00302927	CCT4;ILK-2 T-complex protein 1 subunit delta	634,16	57888	23
8	IPI00027626	CCT6A T-complex protein 1 subunit zeta	274,37	57988	24
9	IPI00855912	PUF60 Isoform 6 of Poly(U)-binding-splicing factor PUF60	573,96	55364	24
10	IPI00396485	EEF1A1 Elongation factor 1-alpha 1	318,22	50109	18
11	IPI00021428	ACTA1 Actin, alpha skeletal muscle	305,18	42024	20
12	IPI00003865	HSPA8 Isoform 1 of Heat shock cognate 71 kDa protein	472,34	70854	24
13	IPI00007752	TUBB2C Tubulin beta-2C chain	381,44	49799	17
14	IPI00220834	XRCC5 X-ray repair cross-complementing protein 5 (Ku80)	269,6	82652	21
15	IPI00013683	TUBB3 Tubulin beta-3 chain	479,89	50400	18
16	IPI00013475	TUBB2A Tubulin beta-2A chain	372,99	49875	15
17	IPI00645452	TUBB Tubulin, beta	299,59	47736	16
18	IPI00054042	GTF2I Isoform 1 of General transcription factor II-I	435,39	112346	16
19	IPI00180675	TUBA1A Tubulin alpha-1A chain	347,16	50104	16
20	IPI00883857	HNRNPU Isoform Long of Heterogeneous nuclear ribonucleoprotein U	176,35	90528	20
21	IPI00018465	CCT7 T-complex protein 1 subunit eta	307,21	59329	16
22	IPI00382470	HSP90AA1 Isoform 2 of Heat shock protein HSP 90-alpha	274,16	98099	17
23	IPI00479186	PKM2 Isoform M2 of Pyruvate kinase isozymes M1/M2	372,32	57900	17
24	IPI00022744	CSE1L Isoform 1 of Exportin-2	361,08	110346	18
25	IPI00290770	CCT3 T-complex protein 1 subunit gamma isoform b	265,91	60424	17
26	IPI00646917	NUDT21 Cleavage and polyadenylation specificity factor subunit 5	283,73	26211	15
27	IPI00008438	RPS10 40S ribosomal protein S10	167,76	18886	14
28	IPI00014424	EEF1A2 Elongation factor 1-alpha 2	161,74	50438	9
29	IPI00221222	SUB1 Activated RNA polymerase II transcriptional coactivator p15	209,62	14386	10
30	IPI00171903	HNRNPM Isoform 1 of Heterogeneous nuclear ribonucleoprotein M	121,34	77464	15
31	IPI00008433	RPS5 40S ribosomal protein S5	148,29	22862	11
32	IPI00215780	RPS19 40S ribosomal protein S19	146,43	16051	12

33	IPI00304435	NIPSNAP1 Protein NipSnap homolog 1	143,92	33289	13
34	IPI00645948	HMGB1 High-mobility group box 1	138,18	25797	10
35	IPI00011253	RPS3 40S ribosomal protein S3	100,01	26671	13
36	IPI00029744	SSBP1 Single-stranded DNA-binding protein, mitochondrial	209,04	17249	10
37	IPI00012998	MIR1279;CPSF6 Isoform 1 of Cleavage and polyadenylation specificity factor subunit 6	205,66	59173	9
38	IPI00013415	RPS7 40S ribosomal protein S7	109,16	22113	11
39	IPI00219483	SNRNP70 Isoform 2 of U1 small nuclear ribonucleoprotein 70 kDa	96,21	50587	12
40	IPI00020127	RPA1 Replication protein A 70 kDa DNA-binding subunit	247,82	68095	9
41	IPI00027107	TUFM Tu translation elongation factor, mitochondrial precursor	241,55	49843	10
42	IPI00215914	ARF1 ADP-ribosylation factor 1	183,99	20684	9
43	IPI00410693	SERBP1 SERPINE1 mRNA binding protein 1, isoform CRA_d	158,12	50890	10
44	IPI00018350	MCM5 DNA replication licensing factor MCM5	134,17	82233	10
45	IPI00017963	SNRPD2 Small nuclear ribonucleoprotein Sm D2	207,94	13518	7
46	IPI00302925	CCT8 59 kDa protein	192,35	59440	9
47	IPI00440493	ATP5A1 ATP synthase subunit alpha, mitochondrial	191,08	59714	9
48	IPI00221093	RPS17 40S ribosomal protein S17	168,16	15540	7
49	IPI00479058	RPS15 40S ribosomal protein S15	111,23	17029	6
50	IPI00376798	RPL11 Isoform 1 of 60S ribosomal protein L11	93,69	20240	9
51	IPI00023530	CDK5 Cell division protein kinase 5	88,31	33283	10
52	IPI00374054	ENAH Isoform 2 of Protein enabled homolog	76,75	63886	8
53	IPI00221354	FUS Isoform Short of RNA-binding protein FUS	198	53323	7
54	IPI00031556	U2AF2 Isoform 1 of Splicing factor U2AF 65 kDa subunit	136,35	53467	8
55	IPI00028031	ACADVL cDNA FLJ56425, highly similar to Very-long-chain specific acyl-CoAdehydroge	245,83	75162	8
56	IPI00607708	LDHA Isoform 2 of L-lactate dehydrogenase A chain	136,3	36457	7
57	IPI00015911	DLD Dihydrolipoyl dehydrogenase, mitochondrial	118,31	54143	7
58	IPI00465248	ENO1 Isoform alpha-enolase of Alpha-enolase	108,17	47139	7
59	IPI00020436	RAB11B Ras-related protein Rab-11B	107,66	24473	6
60	IPI00290566	TCP1 T-complex protein 1 subunit alpha	76,22	60306	8
61	IPI00299904	MCM7 Isoform 1 of DNA replication licensing factor MCM7	168,77	81257	7
62	IPI00215918	ARF4 ADP-ribosylation factor 4	152,25	20498	7
63	IPI00003362	HSPA5 HSPA5 protein	141,38	72377	7
64	IPI00004416	CHMP2A Charged multivesicular body protein 2a	138,77	25088	6
65	IPI00297779	CCT2 T-complex protein 1 subunit beta	117,62	57452	6
66	IPI00013881	HNRNPH1 Heterogeneous nuclear ribonucleoprotein H	101,61	49198	7
67	IPI00643920	TKT cDNA FLJ54957, highly similar to Transketolase	100,01	68698	6
68	IPI00010720	CCT5 T-complex protein 1 subunit epsilon	87,93	59633	6

69	IPI00438229	TRIM28 Isoform 1 of Transcription intermediary factor 1-beta	81,47	88493	7
70	IPI00018146	YWHAQ 14-3-3 protein theta	77,99	27747	6
71	IPI00219097	HMGB2 High mobility group protein B2	55,33	24019	6
72	IPI00186290	EEF2 Elongation factor 2	51,05	95277	7
73	IPI00935624	LOC100293829 similar to hCG2040565	49,28	13902	5
74	IPI00215965	HNRNPA1 Isoform A1-B of Heterogeneous nuclear ribonucleoprotein A1	191,46	38723	6
75	IPI00005978	SFRS2 Splicing factor, arginine/serine-rich 2	135,01	25461	4
76	IPI00398625	HRNR Hornerin	123,81	282228	3
77	IPI00008964	RAB1B Ras-related protein Rab-1B	105,37	22157	5
78	IPI00396378	HNRNPA2B1 Isoform B1 of Heterogeneous nuclear ribonucleoproteins A2/B1	81,9	37407	6
79	IPI00012493	RPS20 40S ribosomal protein S20	70,98	13364	4
80	IPI00166874	DDX39 DEAD (Asp-Glu-Ala-Asp) box polypeptide 39, isoform CRA_c	69,35	36553	4
81	IPI00027285	SNRPB Isoform SM-B' of Small nuclear ribonucleoprotein-associated proteins B and B'	60	24594	4
82	IPI00016077	GBAS Protein NipSnap homolog 2	51,69	33721	6
83	IPI00012382	SNRPA U1 small nuclear ribonucleoprotein A	47,41	31259	5
84	IPI00419585	PPIA Peptidyl-prolyl cis-trans isomerase A	41,35	18001	6
85	IPI00029267	SNRPB2 U2 small nuclear ribonucleoprotein B''	36	25470	5
86	IPI00027462	S100A9 Protein S100-A9	152,2	13234	4
87	IPI00006034	CRIP2 Cysteine-rich protein 2	149,62	22478	4
88	IPI00940786	MCM3 DNA replication licensing factor MCM3	139,75	90924	3
89	IPI00550821	CPSF7 Isoform 1 of Cleavage and polyadenylation specificity factor subunit 7	130,47	52018	5
90	IPI00215919	ARF5 ADP-ribosylation factor 5	122,76	20517	5
91	IPI00328840	THOC4 THO complex subunit 4	104,95	27541	5
92	IPI00257508	DPYSL2 Dihydropyrimidinase-related protein 2	94,85	62255	5
93	IPI00017617	DDX5 Probable ATP-dependent RNA helicase DDX5	94,28	69105	5
94	IPI00011307	MTHFD2 Bifunctional methylenetetrahydrofolate dehydrogenase/cyclohydrolase, mitoch	94,15	37871	4
95	IPI00027350	PRDX2 Peroxiredoxin-2	81,15	21878	4
96	IPI00298547	PARK7 Protein DJ-1	72,77	19878	3
97	IPI00016339	RAB5C Ras-related protein Rab-5C	69,4	23468	5
98	IPI00013468	BUB3 Isoform 1 of Mitotic checkpoint protein BUB3	68,14	37131	5
99	IPI00019848	HCFC1 Isoform 1 of Host cell factor 1	67,22	208602	5
100	IPI00215920	ARF6 ADP-ribosylation factor 6	66,28	20069	5
101	IPI00643041	RANP1;RAN GTP-binding nuclear protein Ran	61,26	24408	5
102	IPI00000494	RPL5;SNORD21 60S ribosomal protein L5	54,5	34341	5
103	IPI00024933	RPL12 Isoform 1 of 60S ribosomal protein L12	53,6	17808	5
104	IPI00479997	STMN1 Stathmin	50,14	17292	5

105	IPI00031820	FARSA Phenylalanyl-tRNA synthetase alpha chain	43,34	57528	4
106	IPI00903251	- 14 kDa protein	42,29	13606	5
107	IPI00001639	KPNB1 Importin subunit beta-1	153,89	97108	3
108	IPI00026272	HIST1H2AE;HIST1H2AB;HIST1H2AD;HIST1H2AL;HIST1H2AM;HIST1H2AI;HIST1H2A	134,06	14127	3
109	IPI00909841	- cDNA FLJ51435, moderately similar to Cofilin-1	133,63	12451	4
110	IPI00291928	RAB14 Ras-related protein Rab-14	120,74	23882	3
111	IPI00011913	HNRNPA0 Heterogeneous nuclear ribonucleoprotein A0	118,26	30822	4
112	IPI00304692	RBMX Heterogeneous nuclear ribonucleoprotein G	92,76	42306	4
113	IPI00005613	U2AF1 Splicing factor U2AF 35 kDa subunit	82	27854	4
114	IPI00029019	UBAP2L Isoform 2 of Ubiquitin-associated protein 2-like	80,24	103867	4
115	IPI00026271	RPS14 40S ribosomal protein S14	78,9	16263	2
116	IPI00219018	GAPDH Glyceraldehyde-3-phosphate dehydrogenase	75,9	36030	4
117	IPI00848226	GNB2L1 Guanine nucleotide-binding protein subunit beta-2-like 1	73,25	35055	4
118	IPI00182289	RPS29 40S ribosomal protein S29	72,25	6672	3
119	IPI00419880	RPS3A 40S ribosomal protein S3a	67,23	29926	3
120	IPI00009841	EWSR1 Ewing sarcoma breakpoint region 1 isoform 1	62,38	68923	4
121	IPI00449049	PARP1 Poly [ADP-ribose] polymerase 1	60,18	113012	4
122	IPI00010271	RAC1 Isoform A of Ras-related C3 botulinum toxin substrate 1	58,16	21436	3
123	IPI00329625	SNORA5B;TBRG4 cDNA FLJ56153, highly similar to Homo sapiens transforming growt	54,26	71812	3
124	IPI00009822	SRP54 Signal recognition particle 54 kDa protein	49,28	55668	4
125	IPI00017297	MATR3 Matrin-3	46,1	94565	4
126	IPI00872762	SUCLG1 Succinyl-CoA ligase [GDP-forming] subunit alpha, mitochondrial	43,28	36227	4
127	IPI00000874	PRDX1 Peroxiredoxin-1	35,51	22096	4
128	IPI00411639	RPSAP15;SNORA6;SNORA62;RPSA Laminin receptor-like protein LAMRL5	154,98	32975	3
129	IPI00031812	YBX1 Nuclease-sensitive element-binding protein 1	101,25	35903	2
130	IPI00163505	RBM39 Isoform 1 of RNA-binding protein 39	94,73	59343	2
131	IPI00007765	HSPA9 Stress-70 protein, mitochondrial	92,4	73635	3
132	IPI00006932	LUC7L2 cDNA FLJ55988, highly similar to RNA-binding protein Luc7-like 2	86,06	54191	3
133	IPI00025491	EIF4A1 Eukaryotic initiation factor 4A-I	82,12	46125	3
134	IPI00011200	PHGDH D-3-phosphoglycerate dehydrogenase	81,46	56614	2
135	IPI00003949	UBE2N Ubiquitin-conjugating enzyme E2 N	76,53	17127	2
136	IPI00003377	SFRS7 Isoform 1 of Splicing factor, arginine/serine-rich 7	76,36	27350	3
137	IPI00216457	HIST2H2AA4;HIST2H2AA3 Histone H2A type 2-A	63,95	14087	3
138	IPI00012726	PABPC4 Isoform 1 of Polyadenylate-binding protein 4	59,75	70738	3
139	IPI00444262	NCL cDNA FLJ45706 fis, clone FEBRA2028457, highly similar to Nucleolin	56,89	65922	3
140	IPI00022434	ALB Putative uncharacterized protein ALB	56,59	71658	3

141	IPI00026302	RPL31 60S ribosomal protein L31	48,49	14454	3
142	IPI00815732	PAICS phosphoribosylaminoimidazole carboxylase, phosphoribosylaminoimidazole succ	46,66	47928	3
143	IPI00217468	HIST1H1B Histone H1.5	43,06	22566	3
144	IPI00021266	SNORD4A;RPL23A 60S ribosomal protein L23a	41,86	17684	3
145	IPI00220301	PRDX6 Peroxiredoxin-6	40,88	25019	3
146	IPI00306516	TIMM44 Mitochondrial import inner membrane translocase subunit TIM44	40,2	51323	3
147	IPI00470610	PYCR2 Pyrroline-5-carboxylate reductase 2	101,99	33616	2
148	IPI00180954	CIRBP Cold-inducible RNA-binding protein	76,56	18637	2
149	IPI00010153	RPL23 60S ribosomal protein L23	69,47	14856	2
150	IPI00219217	LDHB L-lactate dehydrogenase B chain	64,98	36615	2
151	IPI00216049	HNRNPK;MIR7-1 Isoform 1 of Heterogeneous nuclear ribonucleoprotein K	59,4	50944	2
152	IPI00465028	TPI1;TPI1P1 triosephosphate isomerase 1 isoform 2	59,33	30772	2
153	IPI00025753	DSG1 Desmoglein-1	56,92	113676	2
154	IPI00003923	UMPS Isoform 1 of Uridine 5'-monophosphate synthase	56,5	52189	2
155	IPI00016342	RAB7A Ras-related protein Rab-7a	52,26	23475	2
156	IPI00216744	DCX Isoform 1 of Neuronal migration protein doublecortin	51,97	49816	2
157	IPI00012074	HNRNPR Isoform 1 of Heterogeneous nuclear ribonucleoprotein R	50,49	70899	2
158	IPI00427330	SBDS Ribosome maturation protein SBDS	49,81	28745	2
159	IPI00299571	PDIA6 Isoform 2 of Protein disulfide-isomerase A6	49,47	53867	2
160	IPI00015954	SAR1A GTP-binding protein SAR1a	47,17	22353	2
161	IPI00219757	GSTP1 Glutathione S-transferase P	46,91	23341	2
162	IPI00787945	LOC729774 hypothetical protein, partial	45,6	14072	2
163	IPI00022974	PIP Prolactin-inducible protein	43,28	16562	2
164	IPI00176469	CABC1 Isoform 1 of Chaperone activity of bc1 complex-like, mitochondrial	43,2	71904	2
165	IPI00797126	NACA nascent polypeptide-associated complex subunit alpha isoform a	43,14	94622	2
166	IPI00016610	PCBP1 Poly(rC)-binding protein 1	42,88	37474	2
167	IPI00646304	PPIB Peptidyl-prolyl cis-trans isomerase B	42,66	23728	2
168	IPI00783097	GARS Glycyl-tRNA synthetase	38,17	83087	2
169	IPI00003856	ATP6V1E1 V-type proton ATPase subunit E 1	61,82	26129	1
170	IPI00549972	LIMD2 LIM domain-containing protein 2	61,35	14061	1
171	IPI00011631	ZW10 Centromere/kinetochore protein zw10 homolog	59,59	88773	1
172	IPI00217661	RAVER1 ribonucleoprotein PTB-binding 1	58,69	79529	1
173	IPI00013396	SNRPC U1 small nuclear ribonucleoprotein C	57,85	17381	1
174	IPI00027547	DCD Dermcidin	55,01	11277	1
175	IPI00411680	PCMT1 Isoform 1 of Protein-L-isoaspartate(D-aspartate) O-methyltransferase	52,04	24635	1
176	IPI00856049	RPL22L1 60S ribosomal protein L22-like 1	48,01	14598	1

177	IPI00029631	ERH Enhancer of rudimentary homolog	47,99	12251	1
178	IPI00006050	FAM115A Isoform 1 of Protein FAM115A	45,77	102061	1
179	IPI00376503	PYCR1 pyrroline-5-carboxylate reductase 1, mitochondrial isoform 2	44,66	33320	1
